# Supplementary material for: Metasurface-based dual-sense circularly polarized antenna for MIMO/full-duplex applications
Source: PLoS One. 2024 Jun 3;19(6):e0304983. doi: 10.1371/journal.pone.0304983 (PMC11146718; doi:10.1371/journal.pone.0304983)
Supplement: Attachment — Submitted filename: Response to Reviewers.docx [file pone.0304983.s001.docx]

**Original Manuscript ID:** PONE-D-23-32861

**Original Article Title:** “Wideband and high-isolation circularly polarized antenna for MIMO/full-duplex applications”

**To:** Reviewer

**Re:** Response to reviewer

Dear Reviewer,

We appreciate you for your precious time in reviewing our paper and providing valuable comments. It was your valuable and insightful comments that led to possible improvements in the current version. The authors have carefully considered the comments and tried our best to address every one of them.

We are uploading our point-by-point response to the comments, an updated manuscript with red highlighting indicating changes, and a manuscript without track changes.

Best regards,

**Reviewer 1:** Author presented a paper in a very professional way. Author can add some key points to highlight the novelty of research work. Author should read the paper carefully for grammatical errors. Formatting must be as per the journal requirements*.*

**Author response:** The author would like to thank the Reviewer for the constructive comment. The paper has been reorganized for a better demonstration of the contribution of the proposed work.

Generally, instead of using a conventional MS-based antenna, the proposed work uses asymmetric MS. By doing that, the 2-element antenna can be designed with smaller element spacing while achieving comparable operating BW. This has been thoroughly demonstrated in Sections “Single-element design” and “Two-element design”. In addition, the comparison with the related works also demonstrates that the proposed design achieves wideband and high gain isolation with small element spacing. Thus, the authors believe that the presented work has enough contribution for publication in Plos One.

**Author action:** The paper is reorganized with further demonstration of the contribution of the proposed work. In addition, grammatical errors and formatting have also been thoroughly checked.

**Reviewer 2:** *The author reports “Wideband and high-isolation circularly polarized antenna for MIMO/full-duplex applications”. However, there are few queries/recommendations need to be incorporated which are given below.*

***Concern # 1:*** *The relevance of the design is not clear. What is the novelty of the design?*

**Author response:** By the Reviewer’s comment, the paper is reorganized for a better demonstration of the novelty of the proposed work. Instead of using a conventional MS-based antenna, the proposed work uses asymmetric MS. By doing that, the 2-element antenna can be designed with smaller element spacing while achieving comparable operating BW. This has been thoroughly demonstrated in Sections “Single-element design” and “Two-element design”. In addition, the comparison with the related works also demonstrates that the proposed design achieves wideband and high gain isolation with small element spacing. Thus, the authors believe that the presented work has enough contribution for publication in Plos One.

**Author action:** The paper is reorganized and the contribution is emphasized in Sections “Single-element antenna design” and “Two-element antenna design”.

***Concern # 2:*** *The work need to be supported by equivalent circuit model which is missing.*

**Author response:** Agreed.

**Author action:** More details on the operation of the proposed work are mentioned in the Section “Single-element design”. The equivalent circuit is also added to Figure 1 of the revised manuscript.

***Concern # 3:*** *The proof for measurements need to be justified. The measurement with VNA measurement photograph and far-field measurements in Anechoic Chamber need to be added.*

**Author response:** The measurements in the Anechoic Chamber are not available in our lab, we must measure in the outside company. All measurements are conducted by the staff of the company, and we are not allowed to attend. Thus, photographs with the measurement in the Chamber are not taken.

**Author action:** The measurement photo with VNA is added to Fig. 11 of the revised manuscript.

***Concern # 4:*** *Kindly address the modes used in the design?*

**Author response:** Agreed.

**Author action:** Further discussion about the operating mechanism of the proposed work is mentioned in Paragraph 3, Section “Single-element design” of the revised manuscript.

***Concern # 5:*** *The proposed work does not show any novelty and hence the work cannot be recommended for the journal.*

**Author response:** The reply to this comment can be found in the response of comment # 1.
